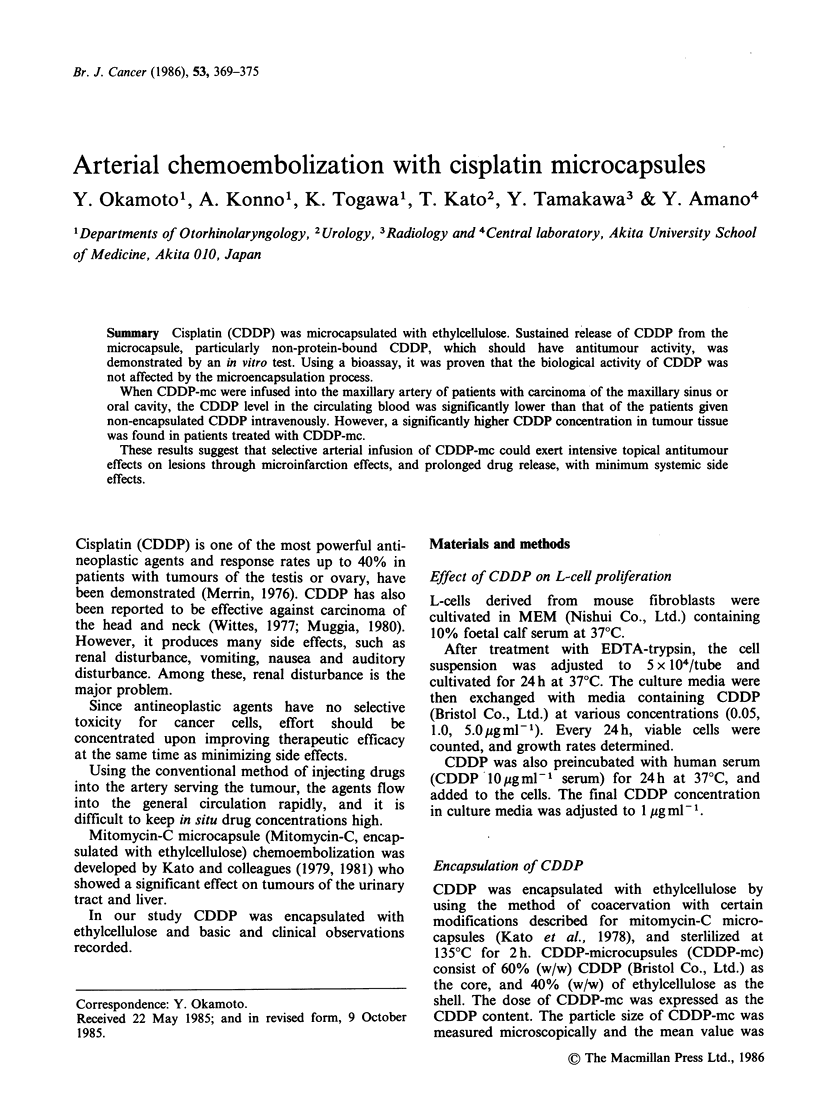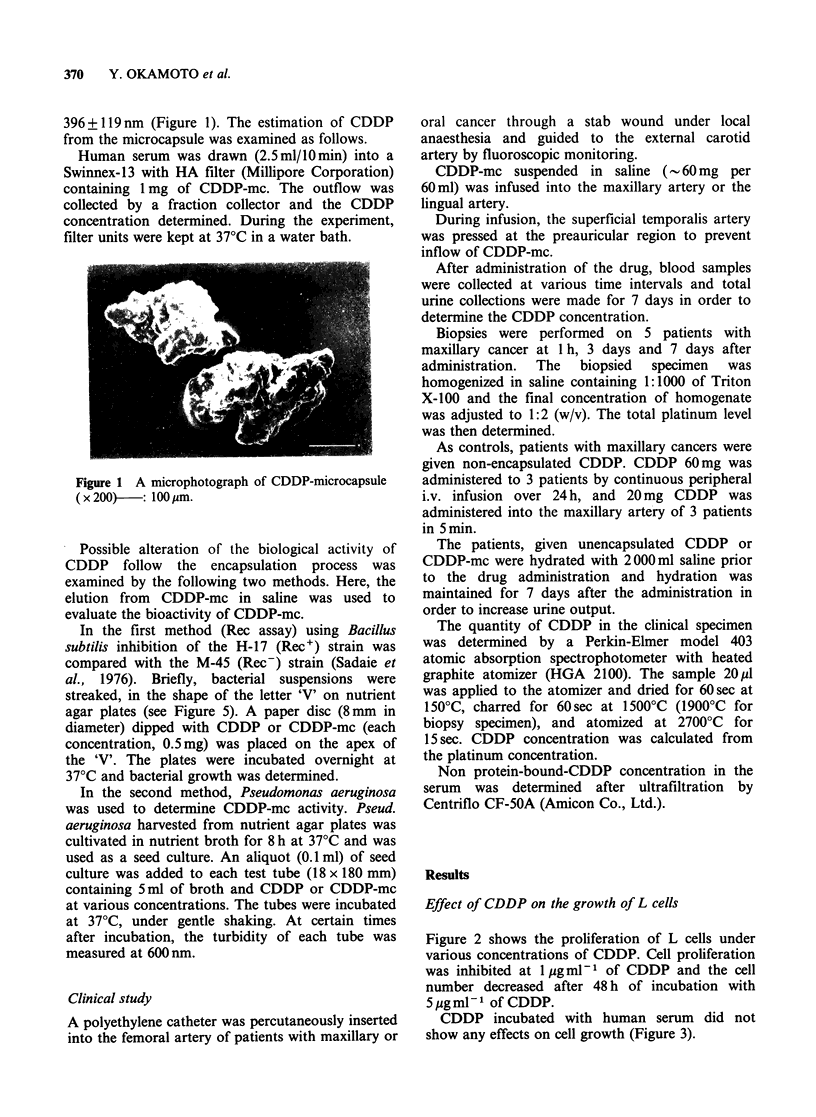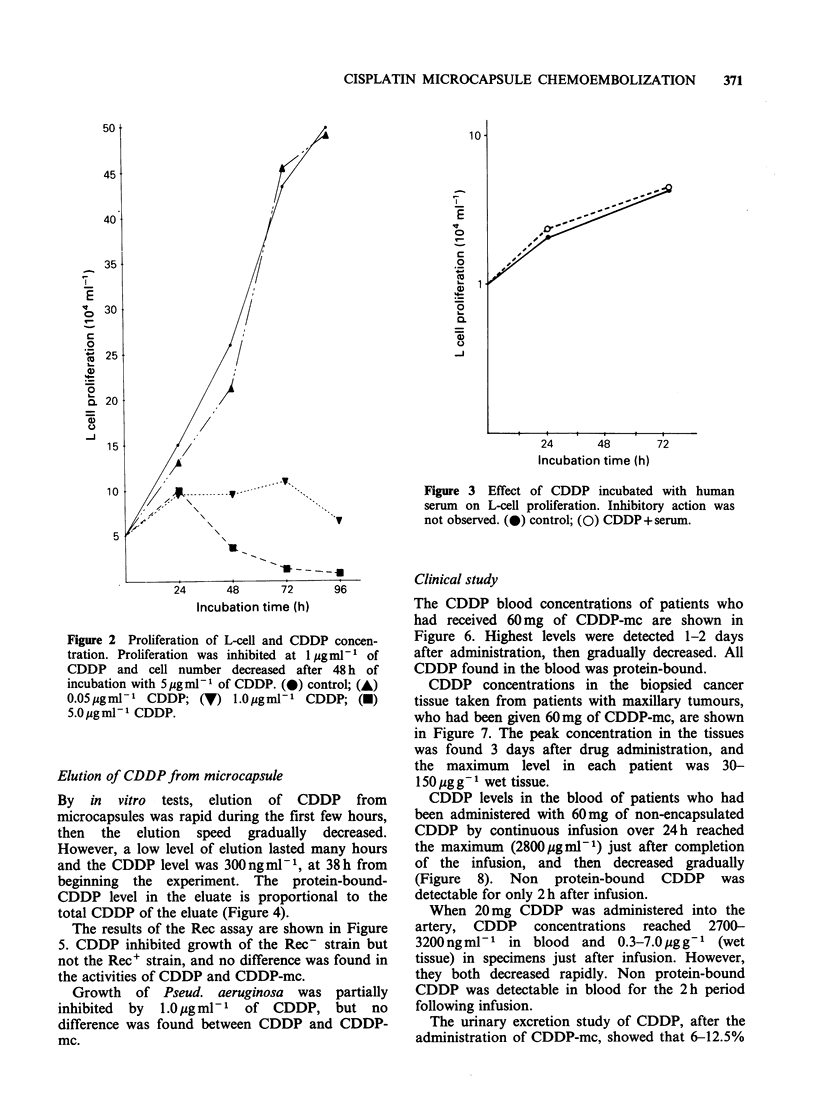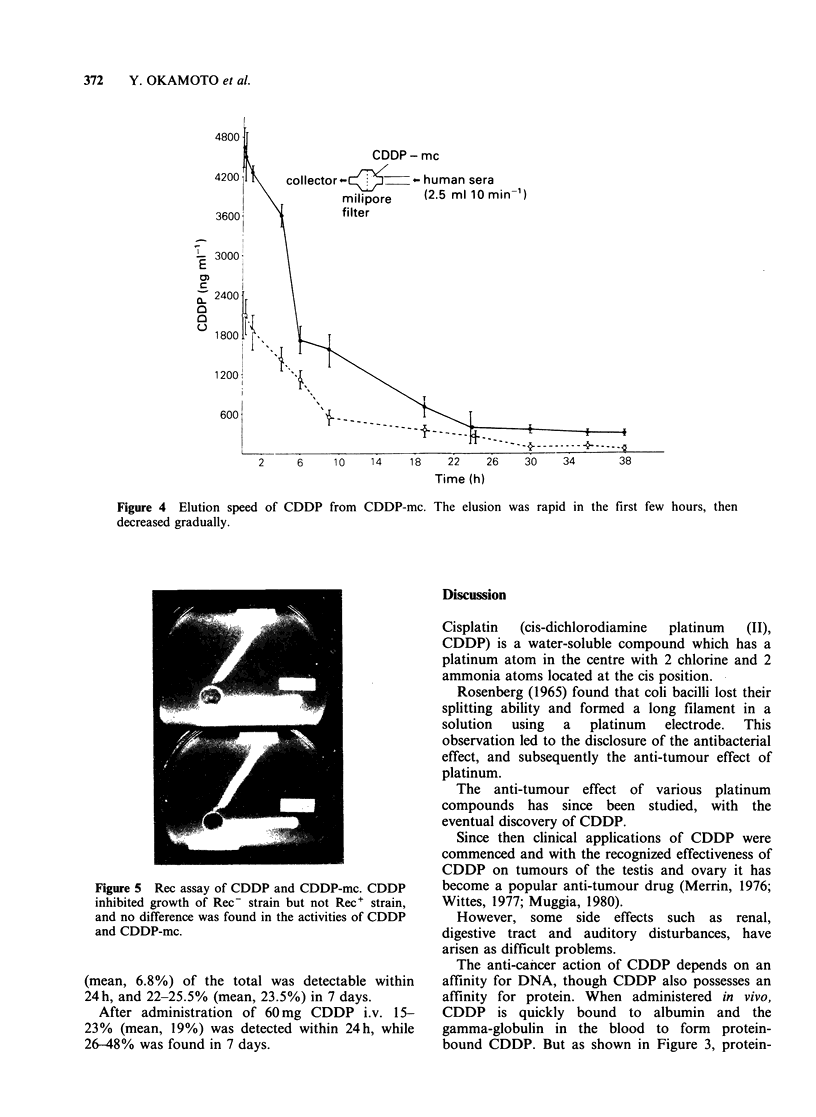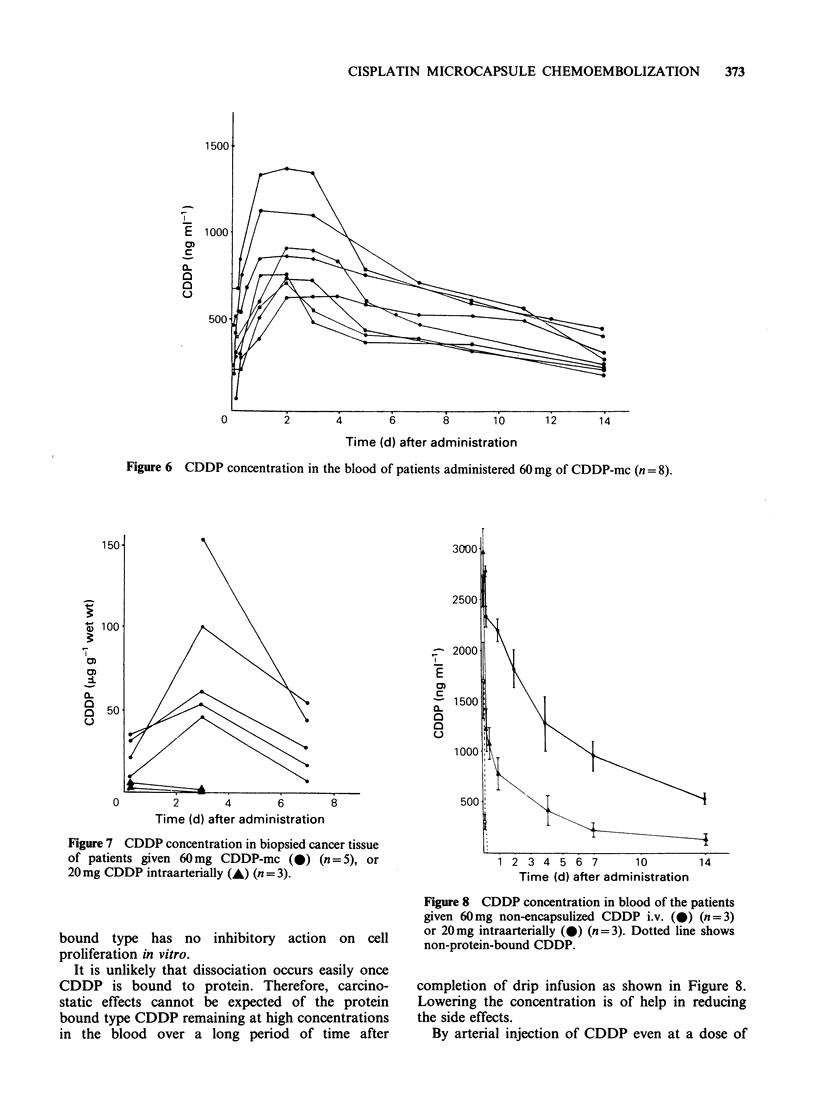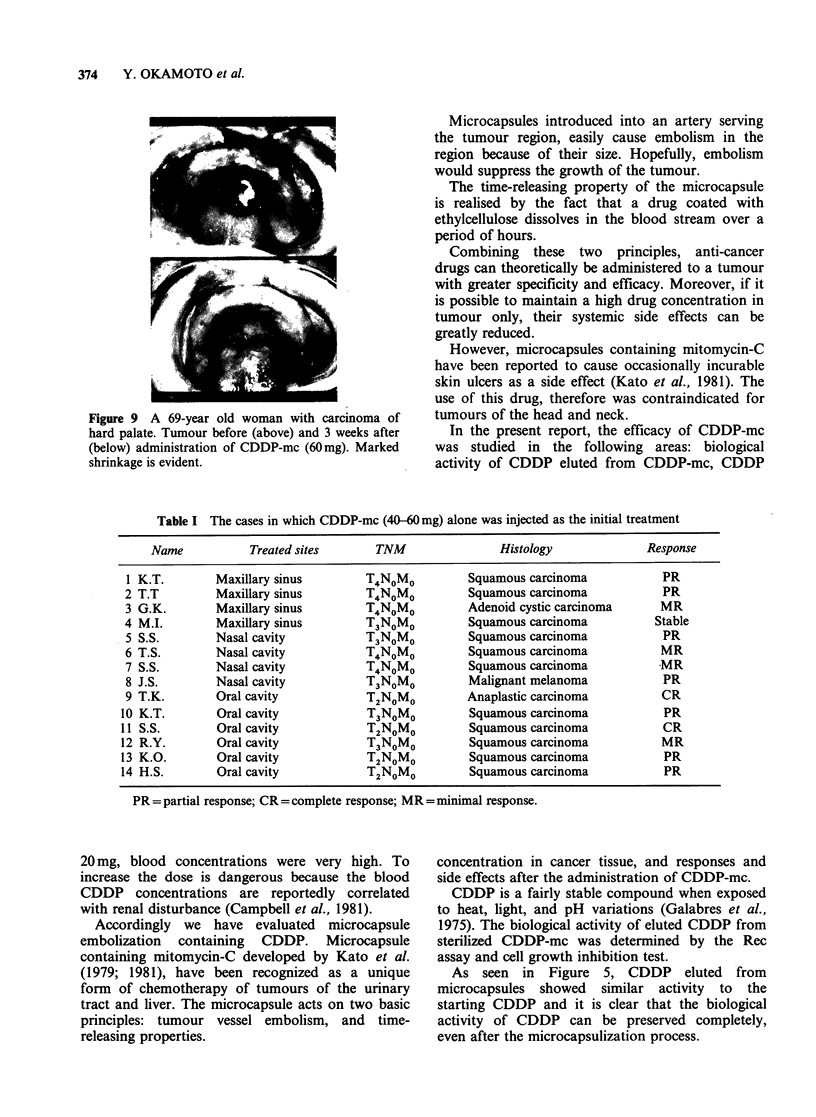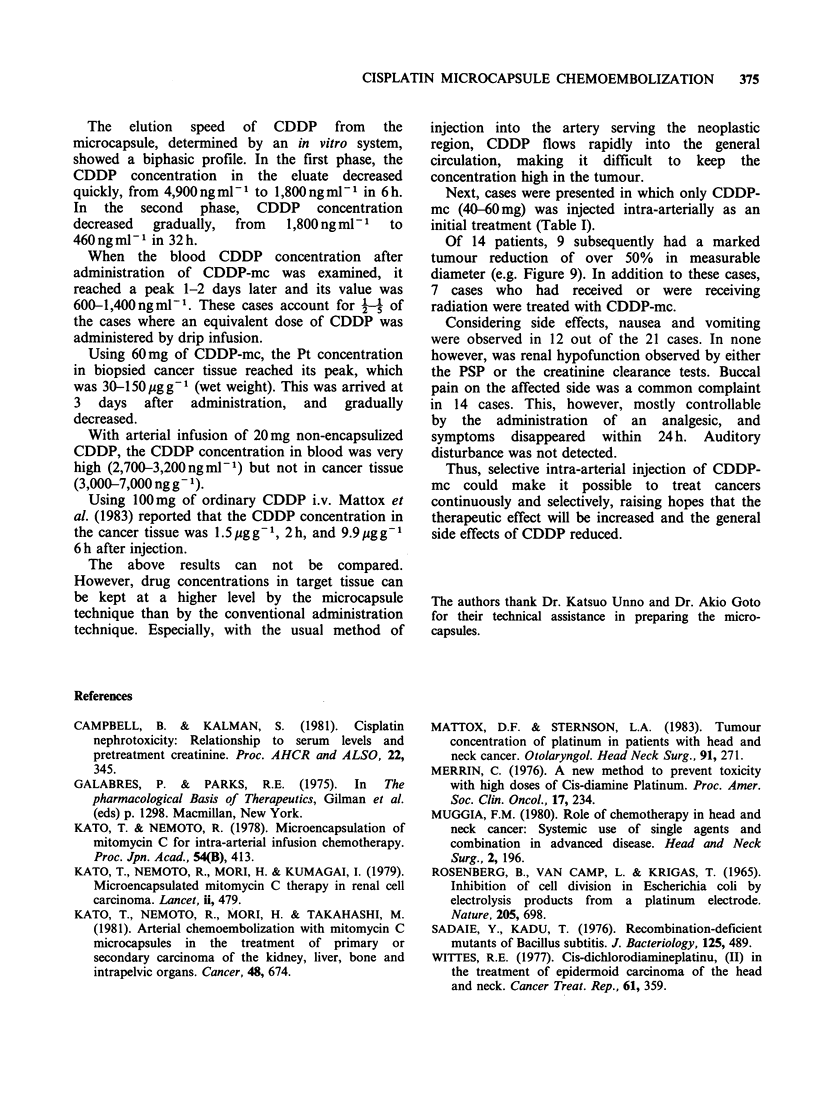# Arterial chemoembolization with cisplatin microcapsules.

**DOI:** 10.1038/bjc.1986.61

**Published:** 1986-03

**Authors:** Y. Okamoto, A. Konno, K. Togawa, T. Kato, Y. Tamakawa, Y. Amano

## Abstract

**Images:**


*Br. J. Cancer* (1986), **53**, 369–375

### Arterial chemoembolization with cisplatin microcapsules

Y. Okamoto[1], A. Konno[1], K. Togawa[1], T. Kato[2], Y. Tamakawa[3] & Y. Amano[4]

[1]*Departments of Otorhinolaryngology,* [2]*Urology,* [3]*Radiology and* [4]*Central laboratory, Akita University School of Medicine, Akita 010, Japan*

**Summary** Cisplatin (CDDP) was microcapsulated with ethylcellulose. Sustained release of CDDP from the microcapsule, particularly non-protein-bound CDDP, which should have antitumour activity, was demonstrated by an *in vitro* test. Using a bioassay, it was proven that the biological activity of CDDP was not affected by the microencapsulation process.

When CDDP-mc were infused into the maxillary artery of patients with carcinoma of the maxillary sinus or oral cavity, the CDDP level in the circulating blood was significantly lower than that of the patients given non-encapsulated CDDP intravenously. However, a significantly higher CDDP concentration in tumour tissue was found in patients treated with CDDP-mc.

These results suggest that selective arterial infusion of CDDP-mc could exert intensive topical antitumour effects on lesions through microinfarction effects, and prolonged drug release, with minimum systemic side effects.

Cisplatin (CDDP) is one of the most powerful antineoplastic agents and response rates up to 40% in patients with tumours of the testis or ovary, have been demonstrated (Merrin, 1976). CDDP has also been reported to be effective against carcinoma of the head and neck (Wittes, 1977; Muggia, 1980). However, it produces many side effects, such as renal disturbance, vomiting, nausea and auditory disturbance. Among these, renal disturbance is the major problem.

Since antineoplastic agents have no selective toxicity for cancer cells, effort should be concentrated upon improving therapeutic efficacy at the same time as minimizing side effects.

Using the conventional method of injecting drugs into the artery serving the tumour, the agents flow into the general circulation rapidly, and it is difficult to keep *in situ* drug concentrations high.

Mitomycin-C microcapsule (Mitomycin-C, encapsulated with ethylcellulose) chemoembolization was developed by Kato and colleagues (1979, 1981) who showed a significant effect on tumours of the urinary tract and liver.

In our study CDDP was encapsulated with ethylcellulose and basic and clinical observations recorded.

Correspondence: Y. Okamoto.
Received 22 May 1985; and in revised form, 9 October 1985.

### Materials and methods

### *Effect of CDDP on L-cell proliferation*

L-cells derived from mouse fibroblasts were cultivated in MEM (Nishui Co., Ltd.) containing 10% foetal calf serum at 37°C.

After treatment with EDTA-trypsin, the cell suspension was adjusted to $5 \times 10^4$/tube and cultivated for 24 h at 37°C. The culture media were then exchanged with media containing CDDP (Bristol Co., Ltd.) at various concentrations (0.05, 1.0, 5.0 $\mu g\, ml^{-1}$). Every 24 h, viable cells were counted, and growth rates determined.

CDDP was also preincubated with human serum (CDDP 10 $\mu g\, ml^{-1}$ serum) for 24 h at 37°C, and added to the cells. The final CDDP concentration in culture media was adjusted to 1 $\mu g\, ml^{-1}$.

### *Encapsulation of CDDP*

CDDP was encapsulated with ethylcellulose by using the method of coacervation with certain modifications described for mitomycin-C microcapsules (Kato *et al.*, 1978), and sterlilized at 135°C for 2 h. CDDP-microcupsules (CDDP-mc) consist of 60% (w/w) CDDP (Bristol Co., Ltd.) as the core, and 40% (w/w) of ethylcellulose as the shell. The dose of CDDP-mc was expressed as the CDDP content. The particle size of CDDP-mc was measured microscopically and the mean value was

© The Macmillan Press Ltd., 1986

396±119 nm (Figure 1). The estimation of CDDP from the microcapsule was examined as follows.

Human serum was drawn (2.5 ml/10 min) into a Swinnex-13 with HA filter (Millipore Corporation) containing 1 mg of CDDP-mc. The outflow was collected by a fraction collector and the CDDP concentration determined. During the experiment, filter units were kept at 37°C in a water bath.

**Figure 1** A microphotograph of CDDP-microcapsule (×200)——: 100 μm.

Possible alteration of the biological activity of CDDP follow the encapsulation process was examined by the following two methods. Here, the elution from CDDP-mc in saline was used to evaluate the bioactivity of CDDP-mc.

In the first method (Rec assay) using *Bacillus subtilis* inhibition of the H-17 ($Rec^+$) strain was compared with the M-45 ($Rec^-$) strain (Sadaie *et al.*, 1976). Briefly, bacterial suspensions were streaked, in the shape of the letter 'V' on nutrient agar plates (see Figure 5). A paper disc (8 mm in diameter) dipped with CDDP or CDDP-mc (each concentration, 0.5 mg) was placed on the apex of the 'V'. The plates were incubated overnight at 37°C and bacterial growth was determined.

In the second method, *Pseudomonas aeruginosa* was used to determine CDDP-mc activity. *Pseud. aeruginosa* harvested from nutrient agar plates was cultivated in nutrient broth for 8 h at 37°C and was used as a seed culture. An aliquot (0.1 ml) of seed culture was added to each test tube (18 × 180 mm) containing 5 ml of broth and CDDP or CDDP-mc at various concentrations. The tubes were incubated at 37°C, under gentle shaking. At certain times after incubation, the turbidity of each tube was measured at 600 nm.

*Clinical study*

A polyethylene catheter was percutaneously inserted into the femoral artery of patients with maxillary or oral cancer through a stab wound under local anaesthesia and guided to the external carotid artery by fluoroscopic monitoring.

CDDP-mc suspended in saline (~60 mg per 60 ml) was infused into the maxillary artery or the lingual artery.

During infusion, the superficial temporalis artery was pressed at the preauricular region to prevent inflow of CDDP-mc.

After administration of the drug, blood samples were collected at various time intervals and total urine collections were made for 7 days in order to determine the CDDP concentration.

Biopsies were performed on 5 patients with maxillary cancer at 1 h, 3 days and 7 days after administration. The biopsied specimen was homogenized in saline containing 1:1000 of Triton X-100 and the final concentration of homogenate was adjusted to 1:2 (w/v). The total platinum level was then determined.

As controls, patients with maxillary cancers were given non-encapsulated CDDP. CDDP 60 mg was administered to 3 patients by continuous peripheral i.v. infusion over 24 h, and 20 mg CDDP was administered into the maxillary artery of 3 patients in 5 min.

The patients, given unencapsulated CDDP or CDDP-mc were hydrated with 2 000 ml saline prior to the drug administration and hydration was maintained for 7 days after the administration in order to increase urine output.

The quantity of CDDP in the clinical specimen was determined by a Perkin-Elmer model 403 atomic absorption spectrophotometer with heated graphite atomizer (HGA 2100). The sample 20 μl was applied to the atomizer and dried for 60 sec at 150°C, charred for 60 sec at 1500°C (1900°C for biopsy specimen), and atomized at 2700°C for 15 sec. CDDP concentration was calculated from the platinum concentration.

Non protein-bound-CDDP concentration in the serum was determined after ultrafiltration by Centriflo CF-50A (Amicon Co., Ltd.).

### Results

*Effect of CDDP on the growth of L cells*

Figure 2 shows the proliferation of L cells under various concentrations of CDDP. Cell proliferation was inhibited at 1 $\mu g\, ml^{-1}$ of CDDP and the cell number decreased after 48 h of incubation with 5 $\mu g\, ml^{-1}$ of CDDP.

CDDP incubated with human serum did not show any effects on cell growth (Figure 3).

**Figure 2** Proliferation of L-cell and CDDP concentration. Proliferation was inhibited at $1\,\mu g\,ml^{-1}$ of CDDP and cell number decreased after 48 h of incubation with $5\,\mu g\,ml^{-1}$ of CDDP. (●) control; (▲) $0.05\,\mu g\,ml^{-1}$ CDDP; (▼) $1.0\,\mu g\,ml^{-1}$ CDDP; (■) $5.0\,\mu g\,ml^{-1}$ CDDP.

**Figure 3** Effect of CDDP incubated with human serum on L-cell proliferation. Inhibitory action was not observed. (●) control; (○) CDDP+serum.

*Elution of CDDP from microcapsule*

By *in vitro* tests, elution of CDDP from microcapsules was rapid during the first few hours, then the elution speed gradually decreased. However, a low level of elution lasted many hours and the CDDP level was $300\,ng\,ml^{-1}$, at 38 h from beginning the experiment. The protein-bound-CDDP level in the eluate is proportional to the total CDDP of the eluate (Figure 4).

The results of the Rec assay are shown in Figure 5. CDDP inhibited growth of the $Rec^-$ strain but not the $Rec^+$ strain, and no difference was found in the activities of CDDP and CDDP-mc.

Growth of *Pseud. aeruginosa* was partially inhibited by $1.0\,\mu g\,ml^{-1}$ of CDDP, but no difference was found between CDDP and CDDP-mc.

*Clinical study*

The CDDP blood concentrations of patients who had received 60 mg of CDDP-mc are shown in Figure 6. Highest levels were detected 1–2 days after administration, then gradually decreased. All CDDP found in the blood was protein-bound.

CDDP concentrations in the biopsied cancer tissue taken from patients with maxillary tumours, who had been given 60 mg of CDDP-mc, are shown in Figure 7. The peak concentration in the tissues was found 3 days after drug administration, and the maximum level in each patient was 30–$150\,\mu g\,g^{-1}$ wet tissue.

CDDP levels in the blood of patients who had been administered with 60 mg of non-encapsulated CDDP by continuous infusion over 24 h reached the maximum ($2800\,\mu g\,ml^{-1}$) just after completion of the infusion, and then decreased gradually (Figure 8). Non protein-bound CDDP was detectable for only 2 h after infusion.

When 20 mg CDDP was administered into the artery, CDDP concentrations reached 2700–$3200\,ng\,ml^{-1}$ in blood and 0.3–$7.0\,\mu g\,g^{-1}$ (wet tissue) in specimens just after infusion. However, they both decreased rapidly. Non protein-bound CDDP was detectable in blood for the 2 h period following infusion.

The urinary excretion study of CDDP, after the administration of CDDP-mc, showed that 6–12.5%

**Figure 4** Elution speed of CDDP from CDDP-mc. The elusion was rapid in the first few hours, then decreased gradually.

**Figure 5** Rec assay of CDDP and CDDP-mc. CDDP inhibited growth of $Rec^-$ strain but not $Rec^+$ strain, and no difference was found in the activities of CDDP and CDDP-mc.

(mean, 6.8%) of the total was detectable within 24h, and 22–25.5% (mean, 23.5%) in 7 days.

After administration of 60mg CDDP i.v. 15–23% (mean, 19%) was detected within 24h, while 26–48% was found in 7 days.

### Discussion

Cisplatin (cis-dichlorodiamine platinum (II), CDDP) is a water-soluble compound which has a platinum atom in the centre with 2 chlorine and 2 ammonia atoms located at the cis position.

Rosenberg (1965) found that coli bacilli lost their splitting ability and formed a long filament in a solution using a platinum electrode. This observation led to the disclosure of the antibacterial effect, and subsequently the anti-tumour effect of platinum.

The anti-tumour effect of various platinum compounds has since been studied, with the eventual discovery of CDDP.

Since then clinical applications of CDDP were commenced and with the recognized effectiveness of CDDP on tumours of the testis and ovary it has become a popular anti-tumour drug (Merrin, 1976; Wittes, 1977; Muggia, 1980).

However, some side effects such as renal, digestive tract and auditory disturbances, have arisen as difficult problems.

The anti-cancer action of CDDP depends on an affinity for DNA, though CDDP also possesses an affinity for protein. When administered *in vivo,* CDDP is quickly bound to albumin and the gamma-globulin in the blood to form protein-bound CDDP. But as shown in Figure 3, protein-

**Figure 6** CDDP concentration in the blood of patients administered 60 mg of CDDP-mc ($n=8$).

**Figure 7** CDDP concentration in biopsied cancer tissue of patients given 60 mg CDDP-mc (●) ($n=5$), or 20 mg CDDP intraarterially (▲) ($n=3$).

**Figure 8** CDDP concentration in blood of the patients given 60 mg non-encapsulized CDDP i.v. (●) ($n=3$) or 20 mg intraarterially (●) ($n=3$). Dotted line shows non-protein-bound CDDP.

bound type has no inhibitory action on cell proliferation *in vitro*.

It is unlikely that dissociation occurs easily once CDDP is bound to protein. Therefore, carcinostatic effects cannot be expected of the protein bound type CDDP remaining at high concentrations in the blood over a long period of time after completion of drip infusion as shown in Figure 8. Lowering the concentration is of help in reducing the side effects.

By arterial injection of CDDP even at a dose of

**Figure 9** A 69-year old woman with carcinoma of hard palate. Tumour before (above) and 3 weeks after (below) administration of CDDP-mc (60 mg). Marked shrinkage is evident.

Microcapsules introduced into an artery serving the tumour region, easily cause embolism in the region because of their size. Hopefully, embolism would suppress the growth of the tumour.

The time-releasing property of the microcapsule is realised by the fact that a drug coated with ethylcellulose dissolves in the blood stream over a period of hours.

Combining these two principles, anti-cancer drugs can theoretically be administered to a tumour with greater specificity and efficacy. Moreover, if it is possible to maintain a high drug concentration in tumour only, their systemic side effects can be greatly reduced.

However, microcapsules containing mitomycin-C have been reported to cause occasionally incurable skin ulcers as a side effect (Kato *et al.*, 1981). The use of this drug, therefore was contraindicated for tumours of the head and neck.

In the present report, the efficacy of CDDP-mc was studied in the following areas: biological activity of CDDP eluted from CDDP-mc, CDDP

**Table I** The cases in which CDDP-mc (40–60 mg) alone was injected as the initial treatment

| *Name* | *Treated sites* | *TNM* | *Histology* | *Response* |
|---|---|---|---|---|
| 1 K.T. | Maxillary sinus | $T_4N_0M_0$ | Squamous carcinoma | PR |
| 2 T.T | Maxillary sinus | $T_4N_0M_0$ | Squamous carcinoma | PR |
| 3 G.K. | Maxillary sinus | $T_4N_0M_0$ | Adenoid cystic carcinoma | MR |
| 4 M.I. | Maxillary sinus | $T_3N_0M_0$ | Squamous carcinoma | Stable |
| 5 S.S. | Nasal cavity | $T_3N_0M_0$ | Squamous carcinoma | PR |
| 6 T.S. | Nasal cavity | $T_4N_0M_0$ | Squamous carcinoma | MR |
| 7 S.S. | Nasal cavity | $T_4N_0M_0$ | Squamous carcinoma | MR |
| 8 J.S. | Nasal cavity | $T_3N_0M_0$ | Malignant melanoma | PR |
| 9 T.K. | Oral cavity | $T_2N_0M_0$ | Anaplastic carcinoma | CR |
| 10 K.T. | Oral cavity | $T_3N_0M_0$ | Squamous carcinoma | PR |
| 11 S.S. | Oral cavity | $T_2N_0M_0$ | Squamous carcinoma | CR |
| 12 R.Y. | Oral cavity | $T_3N_0M_0$ | Squamous carcinoma | MR |
| 13 K.O. | Oral cavity | $T_2N_0M_0$ | Squamous carcinoma | PR |
| 14 H.S. | Oral cavity | $T_2N_0M_0$ | Squamous carcinoma | PR |

PR = partial response; CR = complete response; MR = minimal response.

20 mg, blood concentrations were very high. To increase the dose is dangerous because the blood CDDP concentrations are reportedly correlated with renal disturbance (Campbell *et al.*, 1981).

Accordingly we have evaluated microcapsule embolization containing CDDP. Microcapsule containing mitomycin-C developed by Kato *et al.* (1979; 1981), have been recognized as a unique form of chemotherapy of tumours of the urinary tract and liver. The microcapsule acts on two basic principles: tumour vessel embolism, and time-releasing properties.

concentration in cancer tissue, and responses and side effects after the administration of CDDP-mc.

CDDP is a fairly stable compound when exposed to heat, light, and pH variations (Galabres *et al.*, 1975). The biological activity of eluted CDDP from sterilized CDDP-mc was determined by the Rec assay and cell growth inhibition test.

As seen in Figure 5, CDDP eluted from microcapsules showed similar activity to the starting CDDP and it is clear that the biological activity of CDDP can be preserved completely, even after the microcapsulization process.

The elution speed of CDDP from the microcapsule, determined by an *in vitro* system, showed a biphasic profile. In the first phase, the CDDP concentration in the eluate decreased quickly, from 4,900 ng ml$^{-1}$ to 1,800 ng ml$^{-1}$ in 6 h. In the second phase, CDDP concentration decreased gradually, from 1,800 ng ml$^{-1}$ to 460 ng ml$^{-1}$ in 32 h.

When the blood CDDP concentration after administration of CDDP-mc was examined, it reached a peak 1–2 days later and its value was 600–1,400 ng ml$^{-1}$. These cases account for $\frac{1}{2}$–$\frac{1}{5}$ of the cases where an equivalent dose of CDDP was administered by drip infusion.

Using 60 mg of CDDP-mc, the Pt concentration in biopsied cancer tissue reached its peak, which was 30–150 $\mu$g g$^{-1}$ (wet weight). This was arrived at 3 days after administration, and gradually decreased.

With arterial infusion of 20 mg non-encapsulized CDDP, the CDDP concentration in blood was very high (2,700–3,200 ng ml$^{-1}$) but not in cancer tissue (3,000–7,000 ng g$^{-1}$).

Using 100 mg of ordinary CDDP i.v. Mattox *et al.* (1983) reported that the CDDP concentration in the cancer tissue was 1.5 $\mu$g g$^{-1}$, 2 h, and 9.9 $\mu$g g$^{-1}$ 6 h after injection.

The above results can not be compared. However, drug concentrations in target tissue can be kept at a higher level by the microcapsule technique than by the conventional administration technique. Especially, with the usual method of injection into the artery serving the neoplastic region, CDDP flows rapidly into the general circulation, making it difficult to keep the concentration high in the tumour.

Next, cases were presented in which only CDDP-mc (40–60 mg) was injected intra-arterially as an initial treatment (Table I).

Of 14 patients, 9 subsequently had a marked tumour reduction of over 50% in measurable diameter (e.g. Figure 9). In addition to these cases, 7 cases who had received or were receiving radiation were treated with CDDP-mc.

Considering side effects, nausea and vomiting were observed in 12 out of the 21 cases. In none however, was renal hypofunction observed by either the PSP or the creatinine clearance tests. Buccal pain on the affected side was a common complaint in 14 cases. This, however, mostly controllable by the administration of an analgesic, and symptoms disappeared within 24 h. Auditory disturbance was not detected.

Thus, selective intra-arterial injection of CDDP-mc could make it possible to treat cancers continuously and selectively, raising hopes that the therapeutic effect will be increased and the general side effects of CDDP reduced.

The authors thank Dr. Katsuo Unno and Dr. Akio Goto for their technical assistance in preparing the microcapsules.

### References

CAMPBELL, B. & KALMAN, S. (1981). Cisplatin nephrotoxicity: Relationship to serum levels and pretreatment creatinine. *Proc. AHCR and ALSO*, **22**, 345.

GALABRES, P. & PARKS, R.E. (1975). In *The pharmacological Basis of Therapeutics*, Gilman *et al.* (eds) p. 1298. Macmillan, New York.

KATO, T. & NEMOTO, R. (1978). Microencapsulation of mitomycin C for intra-arterial infusion chemotherapy. *Proc. Jpn. Acad.*, **54(B)**, 413.

KATO, T., NEMOTO, R., MORI, H. & KUMAGAI, I. (1979). Microencapsulated mitomycin C therapy in renal cell carcinoma. *Lancet*, **ii**, 479.

KATO, T., NEMOTO, R., MORI, H. & TAKAHASHI, M. (1981). Arterial chemoembolization with mitomycin C microcapsules in the treatment of primary or secondary carcinoma of the kidney, liver, bone and intrapelvic organs. *Cancer*, **48**, 674.

MATTOX, D.F. & STERNSON, L.A. (1983). Tumour concentration of platinum in patients with head and neck cancer. *Otolaryngol. Head Neck Surg.*, **91**, 271.

MERRIN, C. (1976). A new method to prevent toxicity with high doses of Cis-diamine Platinum. *Proc. Amer. Soc. Clin. Oncol.*, **17**, 234.

MUGGIA, F.M. (1980). Role of chemotherapy in head and neck cancer: Systemic use of single agents and combination in advanced disease. *Head and Neck Surg.*, **2**, 196.

ROSENBERG, B., VAN CAMP, L. & KRIGAS, T. (1965). Inhibition of cell division in Escherichia coli by electrolysis products from a platinum electrode. *Nature*, **205**, 698.

SADAIE, Y., KADU, T. (1976). Recombination-deficient mutants of Bacillus subtitis. *J. Bacteriology*, **125**, 489.

WITTES, R.E. (1977). Cis-dichlorodiamineplatinu, (II) in the treatment of epidermoid carcinoma of the head and neck. *Cancer Treat. Rep.*, **61**, 359.